# Computational and Biochemical Discovery of RSK2 as a Novel Target for Epigallocatechin Gallate (EGCG)

**DOI:** 10.1371/journal.pone.0130049

**Published:** 2015-06-17

**Authors:** Hanyong Chen, Ke Yao, Xiaoyu Chang, Jung-Hyun Shim, Hong-Gyum Kim, Margarita Malakhova, Dong-Joon Kim, Ann M. Bode, Zigang Dong

**Affiliations:** The Hormel Institute, University of Minnesota, 801 16th Ave NE, Austin, MN, 55912, United States of America; Winship Cancer Institute of Emory University, UNITED STATES

## Abstract

The most active anticancer component in green tea is epigallocatechin-3-gallate (EGCG). Protein interaction with EGCG is a critical step for mediating the effects of EGCG on the regulation of various key molecules involved in signal transduction. By using computational docking screening methods for protein identification, we identified a serine/threonine kinase, 90-kDa ribosomal S6 kinase (RSK2), as a novel molecular target of EGCG. RSK2 includes two kinase catalytic domains in the N-terminal (NTD) and the C-terminal (CTD) and RSK2 full activation requires phosphorylation of both terminals. The computer prediction was confirmed by an *in vitro* kinase assay in which EGCG inhibited RSK2 activity in a dose-dependent manner. Pull-down assay results showed that EGCG could bind with RSK2 at both kinase catalytic domains *in vitro* and *ex vivo*. Furthermore, results of an ATP competition assay and a computer-docking model showed that EGCG binds with RSK2 in an ATP-dependent manner. In RSK2^+/+^ and RSK2^-/-^ murine embryonic fibroblasts, EGCG decreased viability only in the presence of RSK2. EGCG also suppressed epidermal growth factor-induced neoplastic cell transformation by inhibiting phosphorylation of histone H3 at Ser10. Overall, these results indicate that RSK2 is a novel molecular target of EGCG.

## Introduction

For thousands of years, tea has been the most widely consumed beverage in the world. Historically, tea has been credited with various beneficial health effects, including medicinal efficacy in the prevention and treatment of numerous diseases [1, 2]. A number of epidemiological studies have shown that tea exerts cancer preventive activity at a variety of organ sites, including skin, lung, esophagus, colon, and pancreas [3, 4]. The polyphenols from green and black tea, epigallocatechin-3-gallate (EGCG) and theaflavins, respectively, are generally considered to be the most active and effective components of tea for inhibiting carcinogenesis [5]. EGCG is the major polyphenol in green tea and may account for 50 to 80% of the total catechins. A cup of green tea contains 100–200 mg of EGCG [6]. Previous reports showed that tea polyphenols inhibited 12-*O*-tetradecanoyl-phorbol-13-acetate (TPA) and epidermal growth factor (EGF)-induced cell transformation [7]. Several proteins that can directly bind with EGCG have been identified, including Bcl-2, AMP-activated protein kinase, the 67-kDa laminin receptor, Ras-GTPase-activating protein SH3 domain-binding protein 1, glucose-regulated protein 78 and vimentin [8–14]. Recently, the human peptidyl prolyl cis/trans isomerase (Pin1), a downstream effecter of oncogenic Neu/Ras signaling, was crystallized with EGCG at 1.9Å resolution [15]. Even though several protein targets have been identified, the precise mechanisms responsible for the reported health effects of EGCG are still not very well understood. Searching for the EGCG “receptor” or high affinity proteins that bind to EGCG is believed to be an important first step in understanding the molecular and biochemical mechanisms of the health effects of tea polyphenols. In our study, computational docking and shape screening methods were used with our in-house database of kinases to screen for targets of EGCG. Interestingly, the results indicated that both the C-terminal (CTD) and N-terminal (NTD) of ribosomal S6 kinase 2 (RSK2) were in the top 10 of the list as targets of EGCG. RSK2 is a member of the p90RSK protein family that is activated by ERK1/2 and PDK1 (phosphoinositide-dependent kinase 1) [16]. RSK2 translocates to the nucleus when activated by growth factors, peptide hormones, or neurotransmitters. Once in the nucleus, RSK2 can phosphorylate various nuclear proteins, including several histones, activating transcription factor 4, p53, and nuclear factor of activated T-cells [17]. Based on its broad substrate specificity, the RSK2 protein is likely to mediate a variety of cellular processes, including proliferation as well as transformation. Our recent study provided evidence indicating that RSK2 plays an important role in cell transformation induced by tumor promoters such as EGF and TPA [18]. Therefore, deciphering the molecular activation mechanism of RSK2 is extremely important for understanding how to control RSK2 activity. In this study, we demonstrated that EGCG directly targets RSK2 at both kinase catalytic domains and inhibits RSK2 kinase activity in a dose-dependent manner. Moreover, we suggest that the inhibition of cell growth and EGF-induced cell transformation requires RSK2. Overall, these results indicate that EGCG is a novel natural compound that effectively suppresses RSK2 activity.

## Materials and Methods

### Computational screening of targets for EGCG

We have constructed an in-house kinase database, which contains about 120 kinase crystal structures derived from the Research Collaboratory for Structural Bioinformatics (RCSB) Protein Data Bank (PDB) [19]. Each structure was prepared using the Protein Preparation Wizard in Schrödinger Suite 2014 [20]. Hydrogen atoms were added consistent with a pH of 7 and water molecules were removed. For each structure, a corresponding binding grid file for docking was generated based on the respective ATP binding pocket. Virtual Screening Workflow from Schrödinger Suite 2014 was used to perform the docking studies to generate a list of binding scores. In addition, a shape screening program also from Schrödinger Suite 2014 was used to screen an additional in-house database comprised of known kinase inhibitors. The shape similarity method was derived from the idea that molecules possessing similar shapes and electrostatic capabilities might exhibit analogous biological activity. Based on this idea, a similarity screening of EGCG and known kinase inhibitors was also performed.

### Reagents

Dulbecco's modified Eagle's medium (DMEM) and fetal bovine serum (FBS) were from Life Technologies, Inc. (Grand Island, NY). Antibodies against β-actin, phosphorylated RSK2 (Thr577) and His G were from Santa Cruz Biotechnology, Inc. (Santa Cruz, CA). The antibody against Xpress was from Invitrogen Corporation (Carlsbad, CA). The 10X kinase buffer and antibodies against RSK2, phosphorylated histone H3 (Ser10), and total histone H3 were from Cell Signaling Technology, Inc. (Beverly, MA). Active RSK2 was from Upstate Biotechnology, Inc. (Charlottesville, VA) and EGCG was a generous gift from Dr. Chi Tang Ho (Rutgers University, Piscataway, NJ).

### Immunoprecipitation and kinase assay

The pcDNA4-Xpress-His-RSK2 expression vectors were transiently introduced into 293 cells. Total cell lysates (200 μg) were used for immunoprecipitation with the Xpress antibody. One half of immunoprecipitated RSK2 was used for confirmation of immunoprecipitation with His G-horseradish peroxidase and visualized by enhanced chemiluminescence. The other half of the RSK2 was used for an *in vitro* kinase assay with EGCG (0, 1, 5, 10 μM) and 1 μg of histone H3 as substrate. Reactions were stopped with 6X SDS sample buffer. Samples were boiled, separated by 15% SDS-PAGE, and visualized by Western blot and antibodies to detect total histone H3 and phosphorylated histone H3 (Ser10).

### Cell culture

The RSK2^+/+^ and RSK2^-/-^ cells are mouse embryonic fibroblasts isolated from The Hormel Institute breeding colonies of Balb/c wildtype (RSK2^+/+^) and knockout (RSK2^-/-^) mice. The isolation of murine embryonic fibroblast protocol was approved by the University of Minnesota, Institutional Animal Care and Use Committee (IACUC) November 11, 2011 and renewed September 9, 2014. RSK2^+/+^ or RSK2^-/-^ mouse embryonic fibroblasts (MEFs) were cultured in DMEM and 10% heat-inactivated FBS in a 37°C, 5% CO_2_ incubator. JB6 Cl41 cells were cultured in MEM and 5% heat-inactivated FBS. MEFs were maintained by splitting at 80 to 90% confluence and media changed every 3 days. Cells were cytogenetically tested and authenticated before freezing and each vial of frozen cells was thawed and maintained for a maximum of 8 weeks.

### Bacterial expression of His-tagged fusion proteins

For expression of the full-length His-fusion RSK2 (RSK2 FL) protein and deletion mutants encompassing the N-terminal or C-terminal kinase domains of RSK2 (RSK2 NTD or RSK2 CTD), the appropriate plasmids (pET46-His-RSK2 and deletion mutants pET46-His-RSK2) were expressed in BL21 *Escherichia coli*. The RSK2 proteins were purified with a protein refolding kit (Novagen, San Diego, CA),

### Pull-down assays

EGCG (2.5 mg) was coupled to CNBr activated Sepharose 4B (GE Healthcare Biosciences, PA) matrix-beads (0.5 g) in 0.5 M NaCl and 40% DMSO (pH 8.3) overnight, at 4°C, according to the manufacturer’s instructions. Active RSK2 (100 ng), lysates from JB6 Cl41 cells (500 μg), purified His-RSK2 FL (100 ng), purified deletion mutant His-RSK2 NTD (100 ng) or mutant His-RSK2 CTD (100 ng) were mixed with EGCG-conjugated Sepharose 4B beads or with Sepharose 4B beads alone as control (50 μL, 50% suspension). Binding was determined by Western blot with anti-RSK2 or anti-His.

### Preparation of RSK2 FL, RSK2 NTD and RSK2 CTD His-fusion proteins

To generate RSK2 FL, RSK2 NTD and RSK2 CTD His-fusion proteins, the BL21 (Stratagene) bacterial strain was used. A single colony for each vector was cultured, and the His-fusion protein was induced by 0.2 mM isopropyl-L-thio-β-D-galactopyranoside treatment at 25°C for 5 h. Soluble His-tagged RSK2 FL, RSK2 NTD or RSK2 CTD was eluted from nickel-nitrilotriacetic acid agarose (Qiagen) with buffer that contained increasing concentrations of imidazole (100 mM and 200 mM), 150 mM NaCl, and 20 mM Tris pH 8.0. Purified proteins were stored at -70°C until used.

### Western blotting

JB6 Cl41 (5 X10^5^) cells were cultured in 10-cm dishes and then starved in serum-free medium for 24 h. The cells were treated with various concentrations of EGCG (0, 10, 20, 30 or 40 μM) or kaempferol (60 μM; positive control) for 1 h before exposure to EGF (10 ng/ml). RSK2^+/+^ or RSK2^-/-^ MEFs were cultured in 10-cm dishes until they reached 80 to 90% confluence. Cells were harvested and proteins extracted in NP40 lysis buffer [50 mM Tris-HCl (pH 8.0), 150 mM NaCl, 0.5% Nonidet P-40] with freezing and thawing. Protein concentration was measured using a protein assay kit (Bio-Rad, Hercules, CA) for Western blotting. Proteins were transferred onto polyvinylidene difluoride membranes and hybridized with antibodies to detect RSK2, β-actin, phosphorylated histone H3 (Ser10), or total histone H3. Antibody binding was performed at 4°C overnight. Protein bands were visualized with a horseradish peroxidase-conjugated secondary antibody and a chemiluminescence detection kit. The blots were treated with stripping buffer [100 mM β-mercaptoethanol, 2% SDS, 62.5 mM Tris-HCl (pH 6.7)] and re-probed with β-actin and histone H3 antibodies as loading controls.

### Isolation of histone proteins

To isolate histone proteins, cells were homogenized in nuclear preparation buffer [10 mM Tris-HCl (pH 7.6), 150 mM NaCl, 1.5 mM MgCl_2_, 0.65% Nonidet P-40, 1 mM phenylmethylsulfonyl fluoride (PMSF), 10 mM NaF, 1 mM sodium orthovanadate and 25 mM β-glycerophosphate]. Nuclei were recovered by centrifugation at 13,000 rpm for 10 min at 4°C. Nuclei were suspended in buffer [10 mM Tris-HCl (pH 7.6), 10 mM NaCl, 3 mM MgCl_2_, 1 mM PMSF, 10 mM NaF, 1 mM sodium orthovanadate and 25 mM β-glycerophosphate]. Nuclei were extracted with 0.4 N H_2_SO_4_ to isolate total histones. The samples were precipitated with trichloroacetic acid (TCA) and then resuspended in double-distilled H_2_O.

### *In silico* modeling of EGCG binding with RSK2

To date, no full-length RSK2 crystal structure has been reported. However, our laboratory has published the respective CTD and NTD crystal structures of RSK2 [21, 22]. For the present study, the crystal structures CTD (PDB ID: 2QR8) and NTD (PDB ID: 3G51) of RSK2 were retrieved from the Protein Data Bank (PDB) and used for the computational study. First, a 3-dimensional (3-D) model of full-length RSK2 was generated using Prime v3.6 of Schrödinger Suite 2014. The model was based on the RSK2 sequence obtained from UniProt (http://www.uniprot.org/) and the crystal structures of RSK2 NTD and CTD. Prime is a powerful and complete tool for generating accurate receptor models for structure level analysis. It provides an easy-to-use interface from sequence to refined structure. The full-length RSK2 homology structure was generated with loop energy minimization and dynamics by using the Impact module from Schrödinger Suite 2014. Molecular dynamics simulations were performed with default options or modified settings. For the default options, a constant temperature (NVT) simulation was run at room temperature with constant dielectric (dielectric constant 1.0) and the OPLS_2005 force field. For the modified settings, we increased the number of MD steps from 100 to 100,000 and as a result, a total 100 ps simulation was performed for the RSK2 homology structure. Following the molecular dynamics simulation, the full-length RSK2 homology structure was prepared under the standard procedure of the Protein Preparation Wizard in Schrödinger Suite 2014. The 30 Å grid for docking was generated based on the ATP binding pocket for both the NTD and CTD of RSK2. EGCG was prepared for docking by default parameters using the LigPrep program from Schrödinger Suite 2014. Then the docking of EGCG and RSK2 was conducted using the Induced Fit docking program of Schrödinger. The program allows EGCG to bind flexibly within the ATP binding pocket. For the Glide docking parameters, the van der Waals scaling of receptors and ligands was set at 0.5 and only the residues within 5.0 Å of ligands were refined. The other options were kept at default parameters and the best 20 poses were retained under the extra precision (XP) mode. In this way, we could obtain the best-docked representative structure.

### MTS assay

RSK2^+/+^ or RSK2^-/-^ MEFs were seeded (5 × 10^3^) into 96-well plates in 100 μl of 10% FBS/DMEM and then incubated with 10 μM EGCG for 24 or 48 h in a 37°C, 5% CO_2_ incubator. CellTiter 96 Aqueous One Solution (20 μl; Promega, Madison, WI) was added and cells incubated for 1 h in a 37°C, 5% CO_2_ incubator. To stop the reaction, 25 μl of a 10% SDS solution were added and absorbance read at 492 and 690 nm using a plate reader (LabSystem Multiskan MS, LabSystems, Helsinki, Finland).

### Anchorage-independent growth assay

JB6 Cl41 cells (8 X 10^3^/well) were suspended in 1 mL basal medium Eagle (BME), 10% FBS and 0.33% agar and plated with various concentrations of EGCG on 3 mL of solidified BME containing 10% FBS and 0.5% agar with the same concentrations of EGCG as on the top layer. Colony number was determined by microscope and the Image-Pro Plus software program (Media Cybernetics, Inc., Bethesda, MD).

### Statistical analysis

All quantitative data are expressed as mean values ± S.D. or S.E. Significant differences were determined by Student t test or one-way ANOVA and a p value < 0.05 was used as the criterion for statistical significance.

## Results

### RSK2 is identified as a target of EGCG

**Fig 1:**
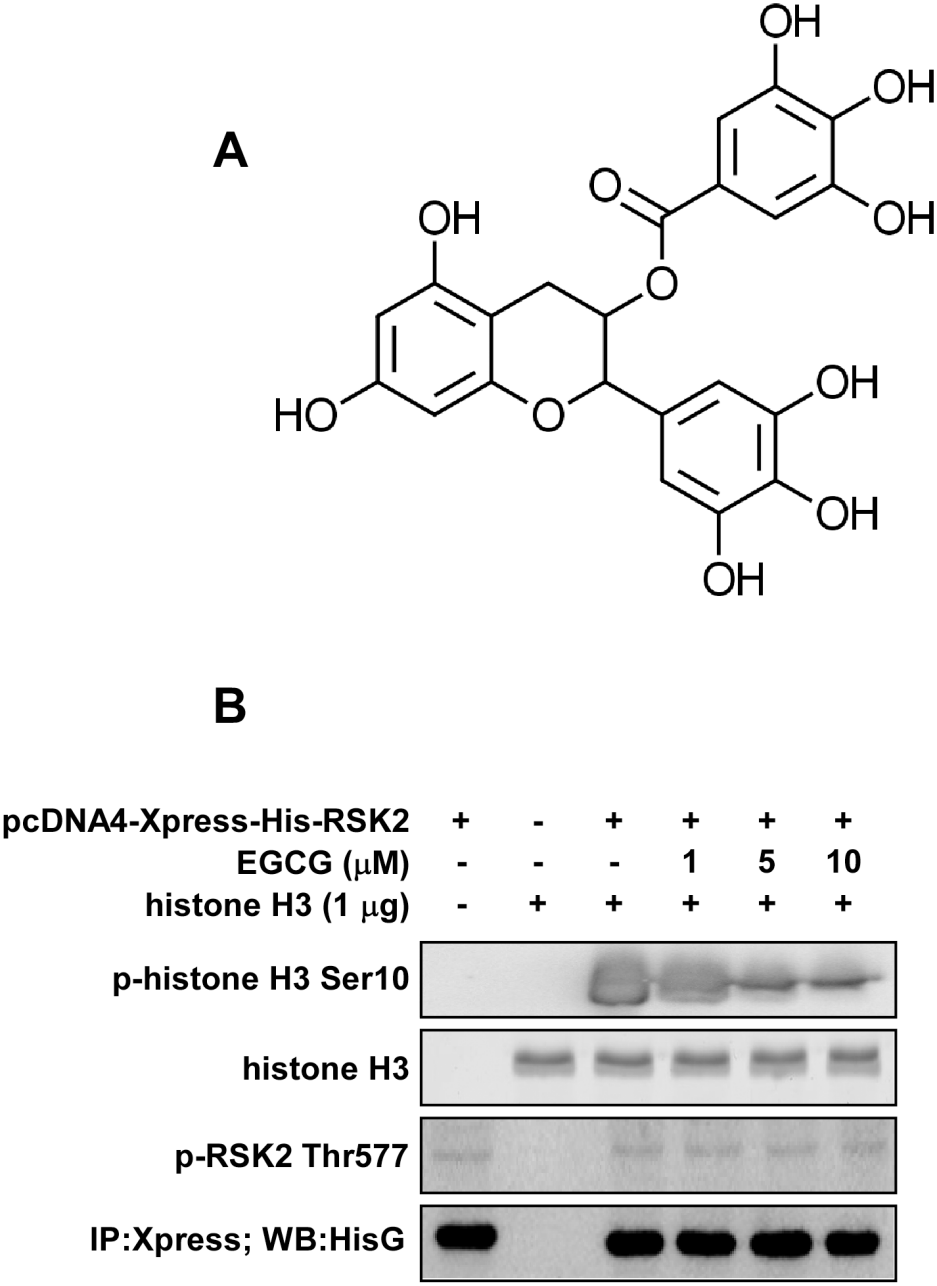
EGCG inhibits RSK2 kinase activity. (A) Chemical structure of EGCG. (B) An immunoprecipitation/kinase assay was performed using different doses of EGCG (0, 1, 5, 10 μM) and 1 μg histone H3 protein as substrate. Results were analyzed by Western blot with antibodies to detect phosphorylated (p-) histone H3 (Ser10), total histone H3, p-RSK2 (Thr577) and His G, respectively. Each assay was performed 3 times and similar results were obtained. Representative blots are shown.

EGCG (Fig 1A) is a major green tea catechin that has been reported to bind with and inhibit the activity of a number of different proteins. Its biological effects appear to be achieved through its modulation of multiple proteins rather than a single target, which supports the concept of poly-pharmacology [23]. To identify additional novel molecular targets of EGCG, a computational docking and screening method was performed using our in-house kinase database. The results indicated that both the C-terminal and N-terminal of RSK2 were in the top 10 list (Table 1). Additionally, our shape screening results showed that EGCG has a similarity score of 7.6 with two known RSK2 inhibitors, quercitrin [24] and afzelin [25]. Together, these results suggested that RSK2 is a potential target of EGCG.

**Table 1:** Top 10 docking results for protein targets of EGCG.

Kinase	docking score
RSK2_CTD	-13.95
PAK4	-13.38
PKACA	-13.23
RSK2_NTD	-13.17
PKC_beta	-12.93
p70 S6K	-12.58
PYK2	-12.45
LCK	-12.33
ASK1	-12.31
PI3-K_delta	-12.28

The abbreviations used are: PAK4, p21-activated kinase 4; PKACA, cAMP-dependent protein kinase catalytic subunit alpha; PKC_beta, Protein Kinase C-beta; p70 S6K, Ribosomol protein S6 kinase, 70kDa, polypeptide 1; PYK2, Protein tyrosine kinase 2 beta; LCK, Leukocyte C-terminal Src kinase; ASK1, Apoptosis signal-regulating kinase 1; PI3-K_delta, Phosphatidylinositol 4,5-bisphosphate 3-kinase catalytic subunit delta.

### EGCG inhibits RSK2 kinase activity

After activation, RSK2 translocates to the nucleus and phosphorylates various nuclear proteins to mediate a numerous cellular processes. One of its well-known phosphorylation substrates is histone H3 [26]. In order to determine whether EGCG affects the ability of RSK2 to phosphorylate histone H3, we conducted an immunoprecipitation-kinase activity assay. The full-length RSK2 was transfected into 293 cells and RSK2 proteins were immunoprecipitated with an Xpress-tagged antibody. The precipitates were directly subjected to an *in vitro* kinase assay with histone H3 (1 μg) as substrate. Treatment with EGCG resulted in a dose-dependent inhibition of histone H3 Ser10 phosphorylation (Fig 1B, upper panel). The active RSK2 protein level was confirmed by antibodies to detect phosphorylated RSK2 (Thr577) and His G (Fig 1B, lower two panels). These results showed that EGCG could prevent optimal phospho-transfer to substrate proteins, such as histone H3, and indicated that EGCG could be an inhibitor of RSK2.

### EGCG binds with both the NTD and CTD catalytic domains of RSK2

**Fig 2:**
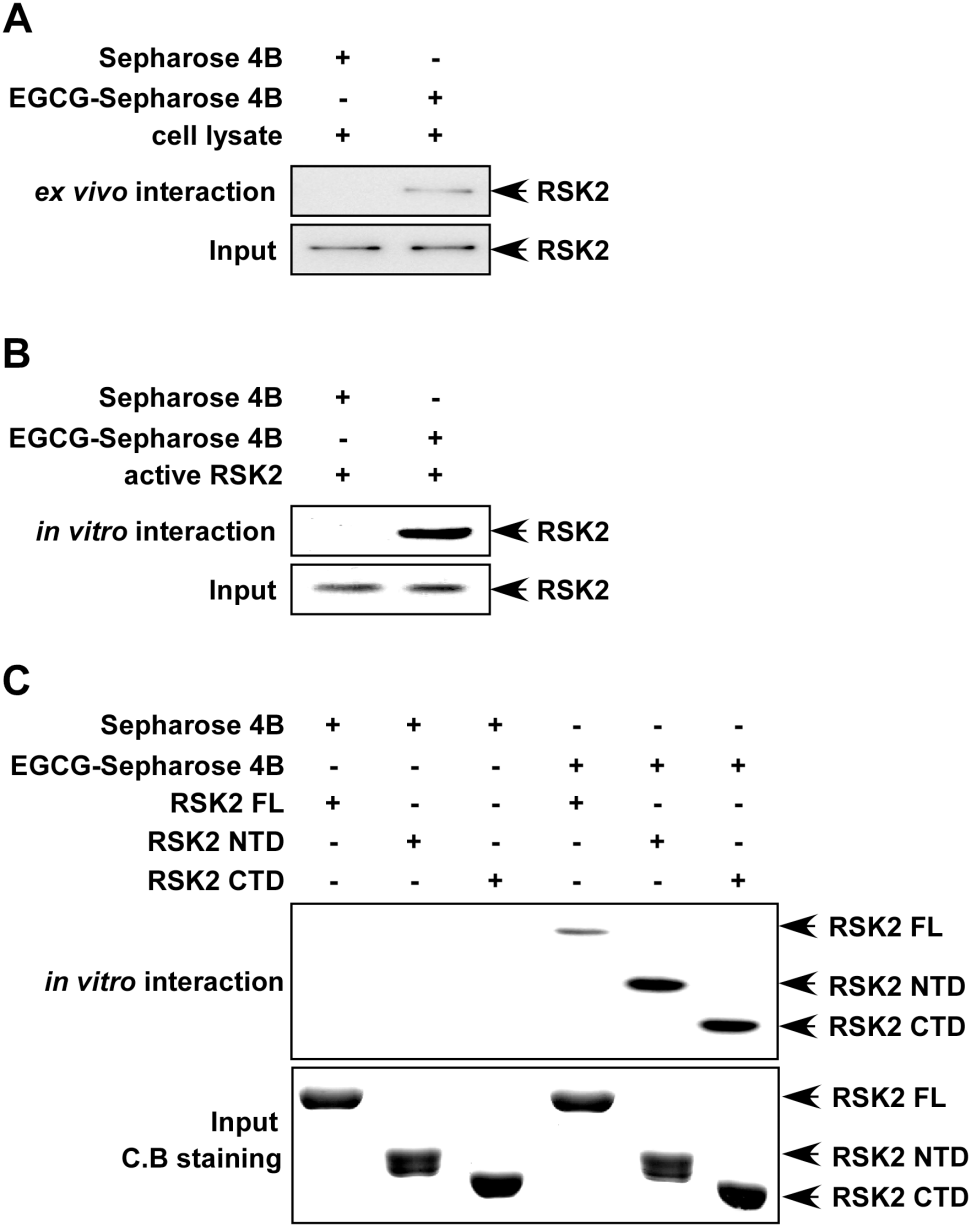
EGCG binds with RSK2 in cells (*ex vivo)* and *in vitro*. (A) EGCG binds with RSK2 *ex vivo*. Whole lysates from JB6 Cl41 cells (500 μg) were incubated with 50 μL of EGCG Sepharose 4B beads (50% slurry) for 2 h at 4°C. Beads were washed and RSK2 binding was visualized by Western blotting with an RSK2 antibody. (B) EGCG binds with active RSK2. Active RSK2 (100 ng) was mixed with EGCG-conjugated Sepharose 4B beads or with Sepharose 4B beads alone and the pulled-down proteins were analyzed by Western blot with an RSK2 antibody. (C) His-tagged full-length (FL) RSK2 fusion proteins or RSK2 deletion mutants (RSK2 NTD or RSK2 CTD) bind with EGCG. A bacterial expressed His-tagged full-length (FL) RSK2 fusion protein or deletion mutant RSK2 NTD or RSK2 CTD (100 ng) was used to examine binding with EGCG Sepharose 4B beads. The bound proteins were visualized by Western blotting with anti-His. The purified His-tagged fusion proteins were stained with Coomassie blue (C.B.). Each assay (A-C) was performed three times. Similar results were obtained and representative blots are shown.

RSK2 is a serine/threonine kinase containing two distinct catalytically functional kinase domains [27]. The C-terminal domain (CTD) phosphorylates the linker region and regulates the N-terminal domain (NTD), which then phosphorylates various substrates [28]. Our previous study revealed that Ser227 phosphorylation in the RSK2 NTD resulted in increased activity toward the S6 peptide both *ex vivo* and *in vitro* [21]. Mutation of tyrosine 707 to alanine (Y707A) in RSK2 resulted in constitutive activation of the CTD [22, 29]. Thus both the NTD and CTD catalytic domains appear to be essential for RSK2 function. In order to determine how EGCG inhibits RSK2 kinase activity, we first investigated whether EGCG could bind with RSK2. To accomplish this, we conjugated EGCG with CNBr-Sepharose 4B beads and conducted a pull-down assay with JB6 Cl41 cells or an active RSK2 protein. Results revealed that EGCG binds with RSK2 *ex vivo* (Fig 2A) and that active RSK2 (100 ng) binds with EGCG-Sepharose 4B beads, but not with Sepharose 4B beads alone (Fig 2B). Furthermore, we conducted a pull-down assay with RSK2 protein fragments, RSK2 FL, RSK2 NTD and RSK2 CTD and observed that each truncated RSK2 protein could bind with CNBr-EGCG beads (Fig 2C). This indicated that both RSK2 NTD and CTD are targets for EGCG.

### EGCG binds with RSK2 in an ATP-competitive manner

**Fig 3:**
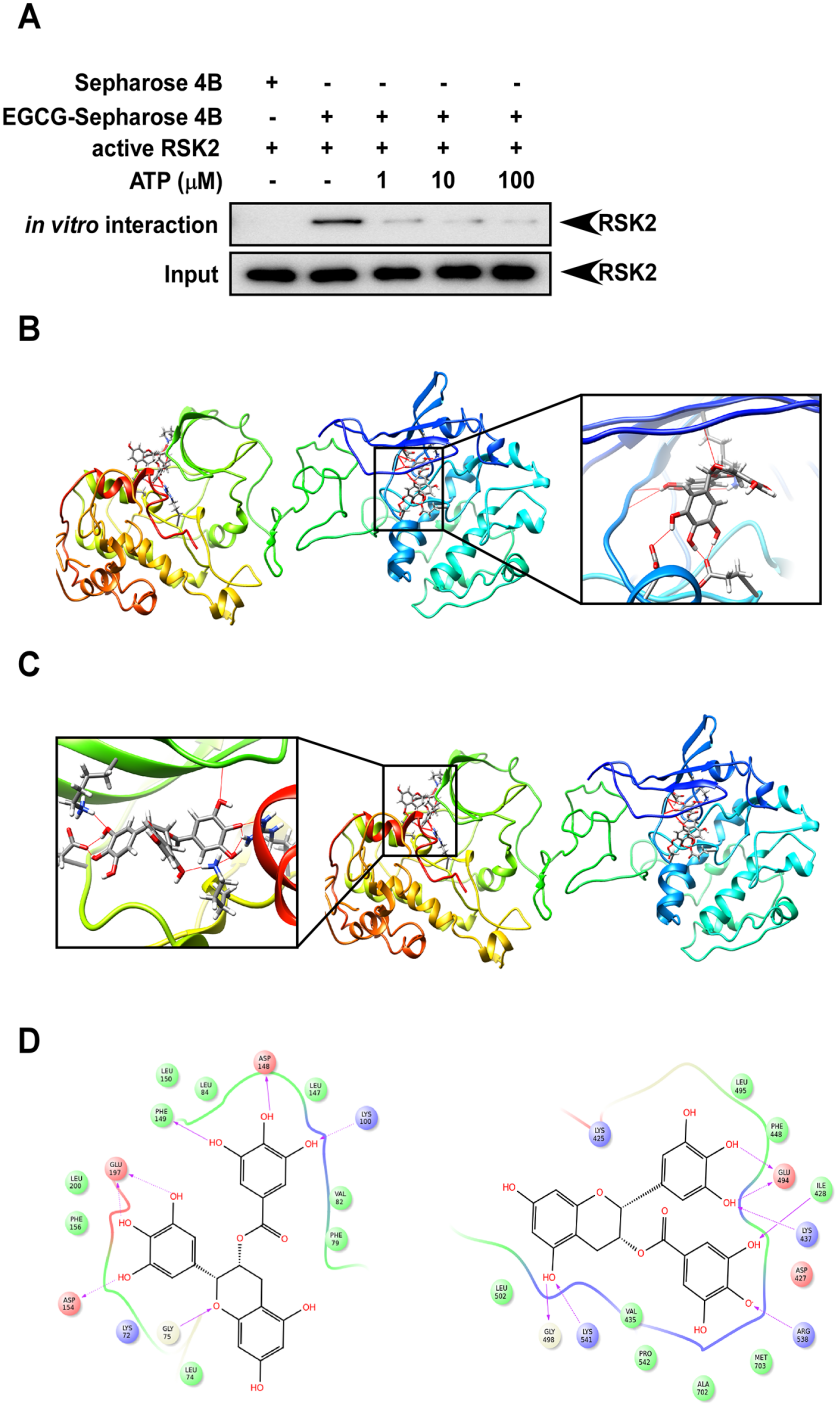
EGCG binds with RSK2 in an ATP-competitive manner. (A) EGCG binds with RSK2 competitively with ATP. Active RSK2 was incubated with 50 μL of EGCG-Sepharose 4B beads (50% slurry) *in vitro* in the presence of increasing concentrations (1–100 μM) of ATP. Beads were washed and RSK2 binding was visualized by Western blotting with an RSK2 antibody. Each assay was performed three times and similar results were obtained and representative Western blots are shown. (B-D) Computer docking models of EGCG binding at the ATP binding pocket of full-length RSK2. Hydrogen bonds are shown as dotted yellow lines in B-C and purple arrows in D. (B) Full-length binding and enlarged view of EGCG binding with RSK2 NTD. (C) Full length binding and enlarged view of EGCG binding with RSK2 CTD. (D) Ligand Interaction Diagram (LID) for EGCG binding with RSK2 (*left*: RSK2 NTD, *right*: RSK2 CTD).

RSK2 phosphorylates the OH group of serines or threonines by adding a phosphate group. Phosphorylation usually results in a functional change in the substrate, such as altering cellular location or its association with other proteins. In RSK2, both the N-terminal and C-terminal catalytic domains contain a glycine-rich stretch of residues in the vicinity of a lysine amino acid, which has been shown to be involved in ATP binding [30]. In the central part of the catalytic domain is a conserved aspartic acid, which is important for the catalytic kinase activity of RSK2 [22]. In order to elucidate the mechanism by which EGCG functions as an RSK2 kinase inhibitor, we conducted an ATP competitive binding assay. Active RSK2 was incubated with 50 μL of EGCG-Sepharose 4B beads (50% slurry) *in vitro* in the presence of increasing concentrations (1–100 μM) of ATP. Beads were washed and RSK2 binding was visualized by Western blotting with an RSK2 antibody. Results showed that EGCG binds with RSK2 in an ATP competitive manner (Fig 3A).

To better understand the interaction between EGCG and RSK2, we constructed a computational docking model of EGCG and the full-length RSK2 homology structure using the Induced Fit Docking program from Schrödinger Suite 2014. In the best score docking models, EGCG fit well into the ATP binding site of RSK2, and showed a good binding mode with the two RSK2 kinase domains. Several important hydrogen bonds are formed between EGCG and the hinge region and the hydrophobic region of both of the RSK2 CTD and NTD (Fig 3B, 3C and 3D), suggesting that EGCG could effectively inhibit RSK2’s kinase activity.

### EGCG inhibits RSK2-mediated cell viability

**Fig 4:**
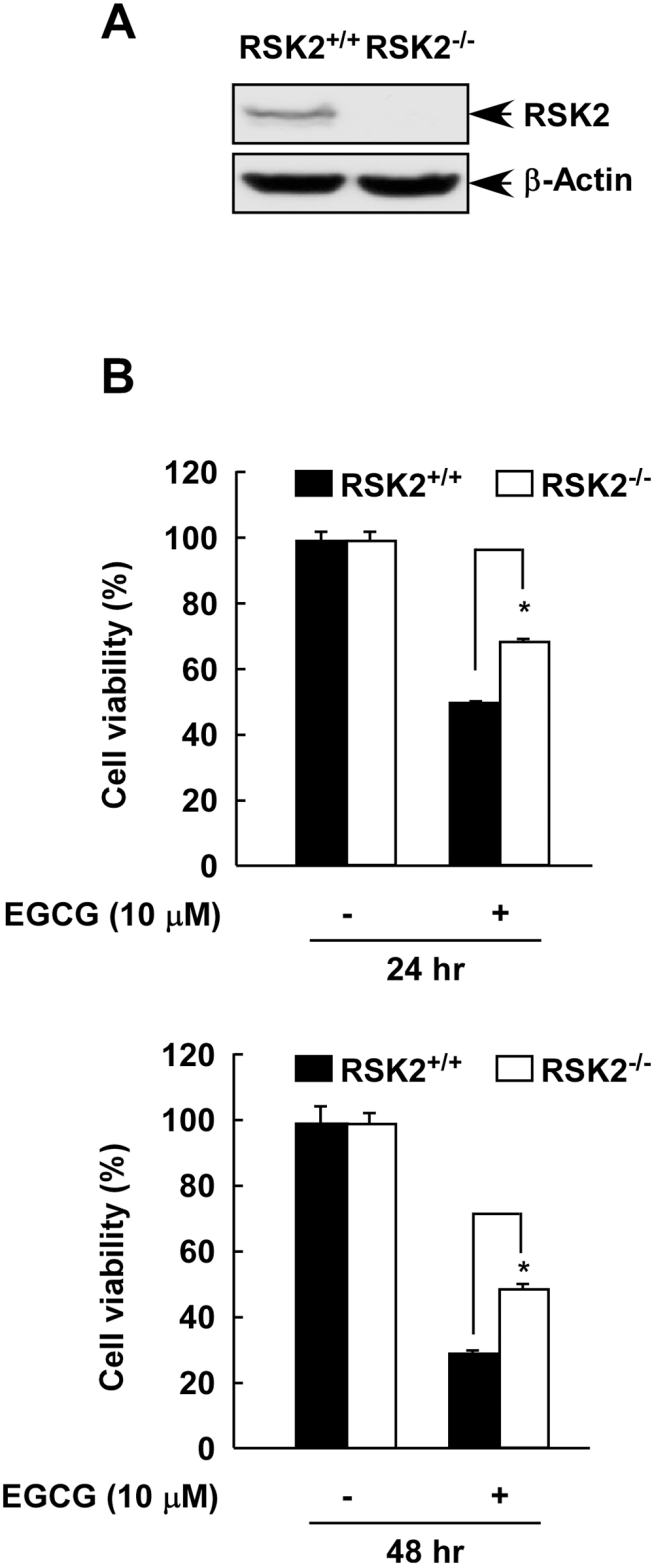
EGCG decreases RSK2-mediated cell viability. (A) RSK2 expression in RSK2^+/+^ and RSK2^-/-^ MEFs. MEFs were cultured until 80–90% confluence was reached. Protein abundance was analyzed by Western blot with an RSK2 antibody. β-Actin was used to verify equal protein loading. Each assay was performed three times and similar results were obtained. Representative Western blots are shown. (B) The effect of EGCG on viability of RSK2^+/+^ and RSK2^-/-^ MEFs. RSK2^+/+^ and RSK2^-/-^ MEFs (5 X 10^3^) were seeded, cultured overnight, and then treated with 10 μM EGCG. Cell viability was measured by MTS assay at the 24 (upper) or 48 (lower) h to assess time-dependent effects. Data are shown as mean values ± S.D. of values obtained from triplicate experiments. Differences were evaluated using the Student’s t-test and the asterisk (*) indicates a significant decrease in viability in RSK2^+/+^ MEFs compared to RSK2^-/-^ MEFs (*p* < 0.05).

The Ras-ERKs signaling pathway regulates cell survival and the MAPK cascades are implicated in the regulation of cell proliferation, survival, growth and motility. RSK2 is an enzyme that acts as a bridge between MAP kinases and a broad range of substrates and has been shown to mediate a variety of cellular processes, including proliferation and transformation [31]. In a comparison of RSK2^+/+^ and RSK2^-/-^ MEFs, Western blot analysis confirmed the absence of the RSK2 protein in RSK2^-/-^ MEFs (Fig 4A). We also found that RSK2^+/+^ and RSK2^-/-^ MEFs grew at a similar rate (S1 Fig). RSK2^+/+^ and RSK2^-/-^ MEFs were used as a model to examine whether the inhibitory effect of EGCG relies on RSK2. We analyzed the viability of RSK2^+/+^ and RSK2^-/-^ MEFs at 24 or 48 h under normal culture conditions with or without 10 μM EGCG. The results indicated that the viability of RSK2^+/+^ MEFs was decreased by about 52% at 24 h and 68% at 48 h by EGCG compared with untreated control cells. In contrast, the viability of RSK2^-/-^ MEFs was decreased by EGCG by about 30% at 24 h and 53% at 48 h compared with untreated control (Fig 4B). These data indicate that EGCG decreased viability more in RSK2^+/+^ MEFs compared to RSK2^-/-^ MEFs suggesting that RSK2 is required for the full inhibitory effect of EGCG.

### EGCG suppresses EGF-induced colony formation mediated through RSK2 in JB6 Cl41 cells

**Fig 5:**
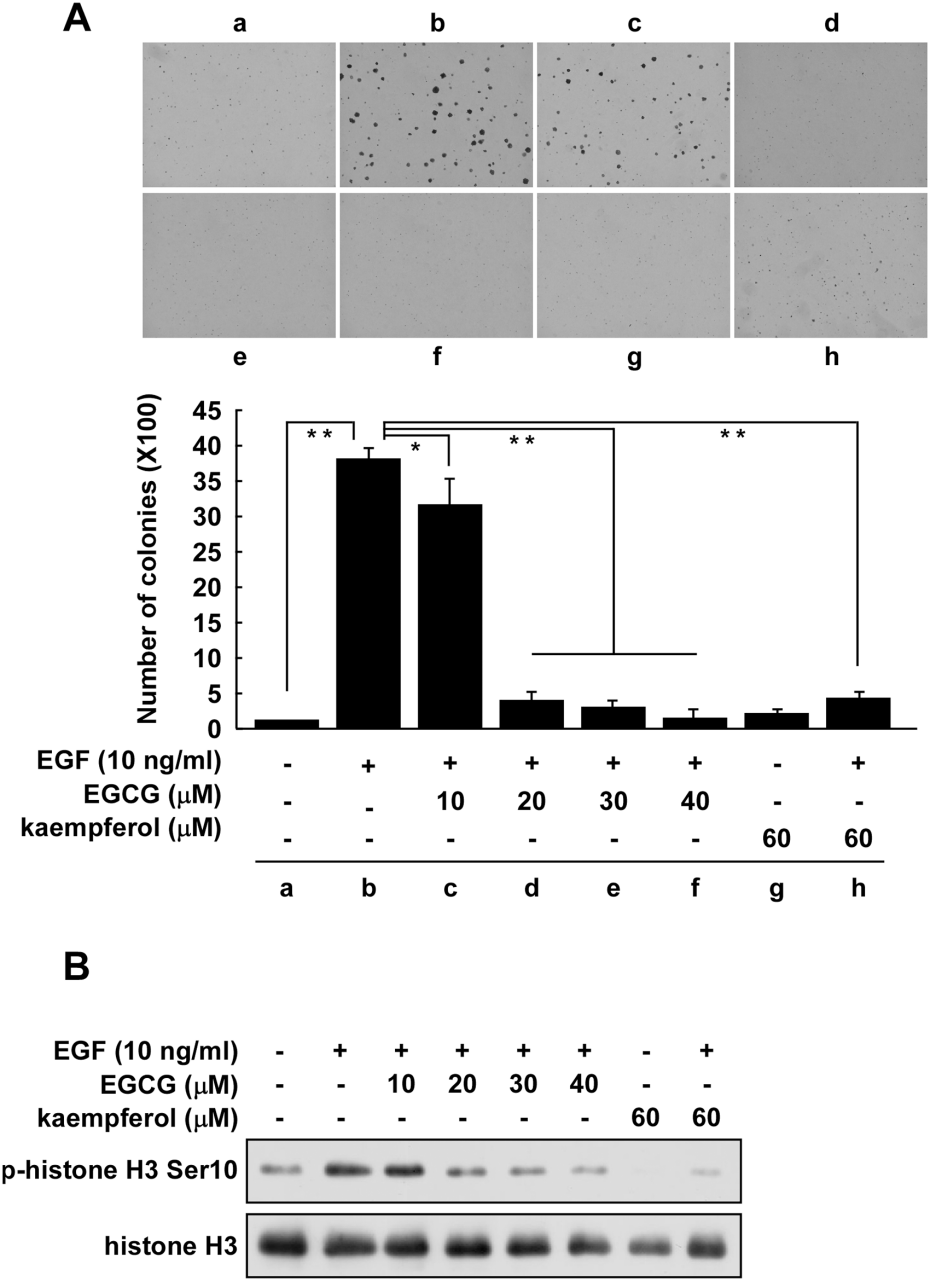
EGCG suppresses EGF-induced colony formation mediated through RSK2 in JB6 Cl41 cells. (A) EGCG suppresses EGF-induced anchorage-independent growth of JB6 Cl41 cells. JB6 Cl41 cells (8 × 10^3^/mL) were exposed to different doses of EGCG in 1 mL of 0.3% BME agar containing 10% FBS with or without 10 ng/mL EGF. Each dose was repeated in triplicate wells. The cultures were maintained in a 37°C, 5% CO_2_ incubator for 10 days and then colonies were counted using a microscope and the Image-Pro PLUS (vs. 4) computer software program. Data are shown as mean values ± S.D. obtained from triplicate experiments. Differences were evaluated using the Student's t test and the asterisks (*, **) indicate avsignificant inhibitory effect of EGCG on colony formation (*p* < 0.05; *p* < 0.01, respectively). (B) EGCG inhibits histone H3 phosphorylation at Ser10. JB6 Cl41cells (5 X 10^5^) were seeded into 10-cm dishes in 5% FBS/MEM and cultured until cells reached 80–90% confluence. The cells were starved for 24 h in 0.1% FBS/MEM. Cells were treated with the indicated dose of EGCG or kaempferol (as positive control) for 1 h and then stimulated with EGF (10 ng/mL) for 15 min and subsequently harvested. Histone proteins were extracted as described in Materials and Methods. The phosphorylation of histone H3 (Ser10) was detected by Western blot. Equal protein loading and transfer were confirmed by stripping and incubating the same membrane with an antibody against total histone H3. Each assay was performed three times and similar results were obtained. Representative blots are shown.

We previously reported that RSK2 promoted anchorage-independent cell growth induced by EGF or TPA [18]. Phosphorylated and total RSK protein levels were increased in a time-dependent manner with EGF or TPA treatment, suggesting that the tumor promoters EGF and TPA activated the ERKs/RSK signaling pathway. As indicated above, RSK2 plays an important role in the inhibition of cell proliferation by EGCG. Therefore, we examined the effect of increasing doses of EGCG (up to 40 μM) on EGF-stimulated colony formation. JB6 Cl41 cells were stimulated for 10 days with EGF (10 ng/mL) with or without different doses of EGCG. The results showed that EGF alone significantly increased colony formation (Fig 5Aa-b). Notably, EGCG inhibited colony formation by 15% at 10 μM (Fig 5Ac) but dramatically inhibited colony formation by 85% at 20 μM (Fig 5Ad-f). Kaempferol, an RSK2 NTD inhibitor, was used as a positive control (Fig 5Ag-h) [28, 32]. Similar results were also observed for the effect of EGCG or kaempferol on colony size (S2 Fig). Thus, EGCG significantly inhibited both the size and number of EGF-induced colonies. We also previously reported that RSK2 is a kinase for EGF-induced histone H3 phosphorylation [32]. Knockout of RSK2 (RSK2^-/-^) totally abolished the phosphorylation of histone H3 at Ser10. Histone H3 phosphorylation at Ser10 by RSK2 is critical for neoplastic cell transformation induced by EGF or TPA [33]. We examined the effect of EGCG on EGF-induced histone H3 phosphorylation at Ser10 and total histone H3 levels in JB6 Cl41 cells. Results indicated that phosphorylated histone H3 (Ser10) was increased by EGF treatment (Fig 5B, lane 2) and was significantly inhibited by treatment with EGCG at 20 μM (Fig 5B, lane 3). Kaempferol was used as a positive control. Overall, these results revealed that EGCG suppresses EGF-induced cell transformation mediated through RSK2.

## Discussion

Cellular kinase-signaling networks are a major regulator of cancer progression and are often involved in pathogenesis. Kinase mutations are relatively common and potent drivers of oncogenesis [34, 35]. Targeting a single kinase has proven successful in some cases, such as the inhibition of EGFR [36]. However, results of this approach have been mixed. Difficulties include rapidly emerging resistance as well as considerable toxicity that can limit dosing to levels that are insufficient for blocking tumor growth [37, 38]. The complexity of cancer has led to recent interest in poly-pharmacological approaches for developing drugs that inhibit kinase activity [23].

The targeted inhibition of multiple kinase signaling pathways by small molecules is becoming an increasingly popular strategy for chemoprevention and chemotherapy [39]. In recent years, multi-target kinase inhibitors have become a focal point of interest for clinicians, with some recently approved for anticancer therapy [40]. This is because highly specific inhibitors of single targets are believed to contribute to drug resistance in cancer patients by stimulating the activation of alternative pathways. Therefore, the broad inhibition of multiple targets, rather than a single specific target, might represent a more complete strategy for the treatment of cancer and other disorders.

Computational methods have been widely applied in biological studies, with the intrinsic advantage of selecting a smaller number of lead compounds in a large database for biological testing, while avoiding expensive and time-consuming experiments [41, 42]. Many computational methodologies can be applied for multiple target inhibitor screening but structure-based molecular docking is the most essential and valuable process [43, 44]. In our laboratory, we have constructed an in-house kinase database containing about 120 kinases and using our IBM supercomputers, we can easily screen lead inhibitors for multiple kinases by using several computational strategies. An inhibitor that could overcome gefitinib resistance in non-small cell lung cancer acting against multiple targets, including PI3-K, ERK1/2, and Aurora A and B, was discovered recently by using these strategies [45].

EGCG, the main and most significant polyphenol in green tea, has shown antioxidant, anti-inflammatory and anti-atherogenic activities [1, 2, 4]. Several direct binding partners of EGCG have been identified, including the intermediate filament protein, vimentin, GRP78, ZAP-70, and Pin1 (at both the WW and PPIase domains) [6, 9–11, 15]. However, the precise mechanisms are still not very well understood. In the present study, computational methods, molecular docking and shape screening, resulted in the identification of 90kDa ribosomal S6 kinase 2 (RSK2) as a new molecular target of EGCG.

Fibroblasts with mutations of the *rsk2* gene failed to exhibit EGF-stimulated phosphorylation of histone H3 at Ser10 [26]. Increased RSK2 levels and phosphorylation of histone H3 have been observed in many tumor cell lines [28, 46]. EGF is known to activate multiple signaling pathways, but only a few key kinases are involved. We previously showed that RSK2 is one of the key signals involved in EGF-induced cell transformation [18]. In that work, we showed that EGF induced RSK2 activation in a time-dependent manner and that ectopic expression of RSK2 in JB6 C41 cells significantly increased anchorage-independent cell transformation and inhibiting or knocking down RSK2 decreased proliferation, anchorage-independent growth and foci formation. We have also previously shown that RSK2 phosphorylates histone H3 at Ser10 when induced by EGF and this phosphorylation was indispensable for neoplastic cell transformation induced by EGF [47]. In the current work, we showed that EGCG could inhibit EGF-induced cell transformation and EGF-induced histone H3 phosphorylation at Ser10 in a manner similar to kaempferol, an RSK2 inhibitor (Figs 4 and 5). Thus we could conclude that EGCG inhibited anchorage-independent cell transformation mediated at least partially through the RSK2/histone H3 signaling pathway.

The CTD is involved in auto-phosphorylation of RSK, and the NTD is responsible for substrate phosphorylation [21, 22]. Therefore both the NTD and CTD catalytic domains appear to be essential for RSK2 substrate activity and function. Our *in vitro* and *ex vivo* pull down assay results indicated that EGCG could bind with RSK2 at both the NTD and CTD in an ATP competitive manner (Figs 2 and 3). We discovered that EGCG competed with ATP for binding with RSK2 (Fig 3A) and dose-dependently inhibited RSK2 phosphorylation of histone H3 at Ser10 (Fig 1B). This suggested that inhibition of the RSK2-histone H3 signaling pathway might be an important mechanism explaining EGCG’s preventive role in carcinogenesis.

Overall, our results showed that EGCG exerted excellent inhibitory effects against EGF-induced cell transformation and proliferation by targeting RSK2 NTD and CTD. Thus, combined our previous reports, EGCG as a poly-pharmacological molecule could be highly beneficial for a new approach to rational drug development.

## Supporting Information

S1 Fig RSK2^+/+^ and RSK2^-/-^ primary MEFs show same proliferation rate. The proliferation rate of RSK2+/+ and RSK2-/- MEFs was measured at the indicated time points by MTS assay. Data are shown as means ± S.D. of values obtained from triplicate experiments.
(TIF)

S2 Fig EGCG suppresses EGF-induced anchorage-independent growth of JB6 Cl41 cells. EGCG significantly inhibits the size of EGF-induced colonies. Using the Image-Pro Premier offline software (Media Cybernetics, Inc., Bethesda, MD), the size of each colony was measured as relative surface area expressed as pixels2. Results are expressed as mean values ± S.D. (*, **, p < 0.05; p < 0.01, respectively) for triplicate experiments.
(TIF)
