# Supplementary figures and images for: Computational and Biochemical Discovery of RSK2 as a Novel Target for Epigallocatechin Gallate (EGCG)
Source: PLoS One. 2015 Jun 17;10(6):e0130049. doi: 10.1371/journal.pone.0130049 (PMC4470687; doi:10.1371/journal.pone.0130049)

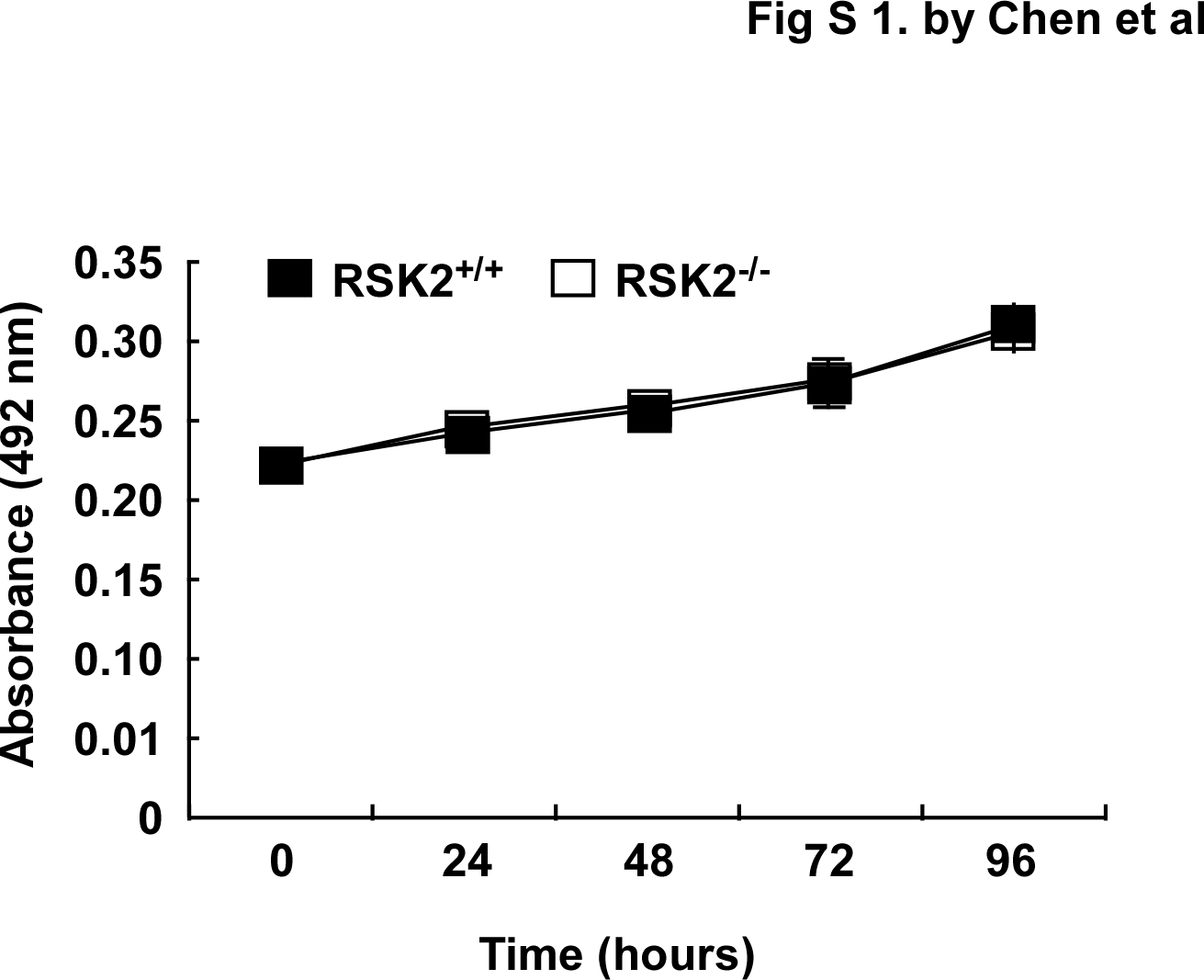

Supplement: S1 Fig — The proliferation rate of RSK2+/+ and RSK2-/- MEFs was measured at the indicated time points by MTS assay. Data are shown as means ± S.D. of values obtained from triplicate experiments. (TIF) [file pone.0130049.s001.tif]

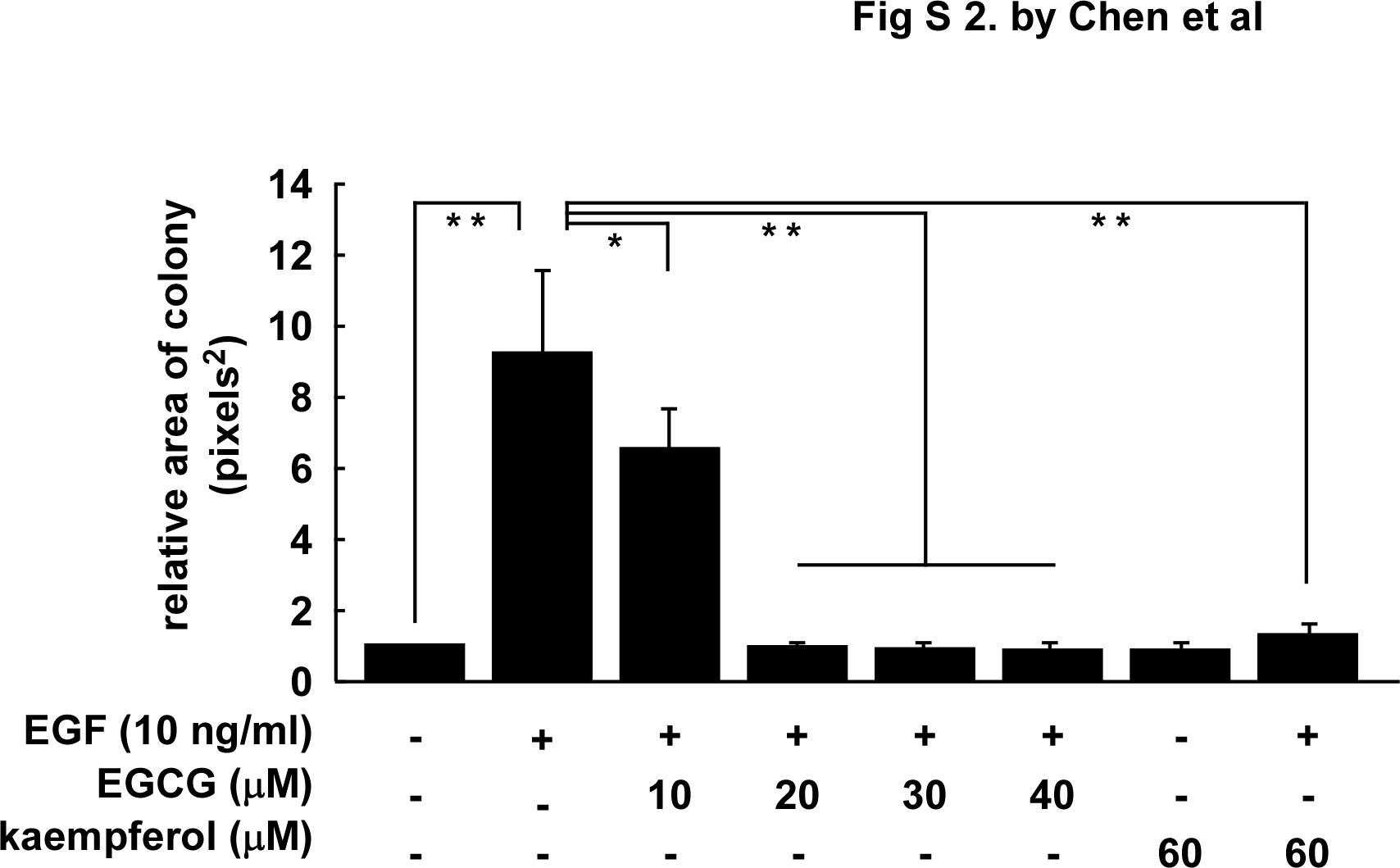

Supplement: S2 Fig — EGCG significantly inhibits the size of EGF-induced colonies. Using the Image-Pro Premier offline software (Media Cybernetics, Inc., Bethesda, MD), the size of each colony was measured as relative surface area expressed as pixels2. Results are expressed as mean values ± S.D. (*, **, p < 0.05; p < 0.01, respectively) for triplicate experiments. (TIF) [file pone.0130049.s002.tif]
